# Zinc oxide nanoparticles application alleviates salinity stress by modulating plant growth, biochemical attributes and nutrient homeostasis in *Phaseolus vulgaris* L

**DOI:** 10.3389/fpls.2024.1432258

**Published:** 2024-09-04

**Authors:** Aayushi Gupta, Rohit Bharati, Jan Kubes, Daniela Popelkova, Lukas Praus, Xinghong Yang, Lucie Severova, Milan Skalicky, Marian Brestic

**Affiliations:** ^1^ Department of Botany and Plant Physiology, Faculty of Agrobiology, Food and Natural Resources, Czech University of Life Sciences Prague, Prague, Czechia; ^2^ Department of Economic Theories, Faculty of Economics and Management, Czech University of Life Sciences Prague, Prague, Czechia; ^3^ Materials Chemistry Department, Institute of Inorganic Chemistry AS CR v.v.i., Husinec-Řež, Czechia; ^4^ Department of Agro-Environmental Chemistry and Plant Nutrition, Faculty of Agrobiology, Food and Natural Resources, Czech University of Life Sciences Prague, Prague, Czechia; ^5^ College of Life Sciences, State Key Laboratory of Crop Biology, Shandong Agricultural University, Taian, China

**Keywords:** beans, foliar spray, nano priming, salinity stress, soil application, ZnO nanoparticles

## Abstract

Salt stress poses a significant challenge to global agriculture, adversely affecting crop yield and food production. The current study investigates the potential of Zinc Oxide (ZnO) nanoparticles (NPs) in mitigating salt stress in common beans. Salt-stressed bean plants were treated with varying concentrations of NPs (25 mg/L, 50 mg/L, 100 mg/L, 200 mg/L) using three different application methods: foliar application, nano priming, and soil application. Results indicated a pronounced impact of salinity stress on bean plants, evidenced by a reduction in fresh weight (24%), relative water content (27%), plant height (33%), chlorophyll content (37%), increased proline (over 100%), sodium accumulation, and antioxidant enzyme activity. Application of ZnO NPs reduced salt stress by promoting physiological growth parameters. The NPs facilitated enhanced plant growth and reduced reactive oxygen species (ROS) generation by regulating plant nutrient homeostasis and chlorophyll fluorescence activity. All the tested application methods effectively mitigate salt stress, with nano-priming emerging as the most effective approach, yielding results comparable to control plants for the tested parameters. This study provides the first evidence that ZnO NPs can effectively mitigate salt stress in bean plants, highlighting their potential to address salinity-induced growth inhibition in crops.

## 1 Introduction

Salt stress is a major abiotic stress that threatens global food security by negatively impacting agriculture and food production. Current estimates suggest that over 20% of cultivated land worldwide is afflicted by salinity stress, which is steadily rising each year (Shrivastava and Kumar, 2015). With this pace, it is estimated that over 50% of the total arable land will be afflicted by salt stress (Jamil et al., 2011). Anthropogenic activities like deforestation, excessive use of fertilizers, poor irrigation practices, salt-contaminated water irrigation, intensive cropping, etc., contribute to salinity stress in soil (Singh, 2019; Kumari et al., 2022). Some natural phenomena, such as rock weathering, irregular rainfall, rising levels of oceans, and high rates of evaporation, further exacerbate salinity stress. Salt stress, primarily caused by higher concentrations of Na^+^ in the soil, impedes the ability of plants to absorb water and nutrients and hampers nutrient balance (Hasegawa et al., 2000). Additionally, salt stress induces toxic radicals like reactive oxygen species (ROS), which negatively affect the photosynthetic machinery of the plants, often causing early leaf senescence and chlorosis (Sudhir and Murthy, 2004). These toxic radicals also damage cell membranes and degrade proteins, enzymes, and pigments, causing plant cellular death and ultimately causing a significant decrease in crop yield (Wu et al., 2014).

To mitigate the salt stress, various measures are employed, including adding organic matter, improving irrigation systems like drip irrigation, fertilization management, and application of NPs. Among these, the application of NPs is gaining attention among researchers for their potential to effectively alleviate numerous abiotic stresses (Juhel et al., 2011).

NPs are characterized by their diminutive particle size within the range of 10-100 nm, with a higher surface area-to-weight ratio, enhancing their reactivity and absorption capability (Ghassemi-Golezani and Abdoli, 2021). Numerous studies have demonstrated that NPs possess the ability to mitigate salt stress and improve plant growth (Avestan et al., 2019; Mahmoud et al., 2019; Pérez-Labrada et al., 2019; Abdoli et al., 2020; Alabdallah and Alzahrani, 2020; Moradbeygi et al., 2020; Zulfiqar and Ashraf, 2021; Ul Haq et al., 2023). Among the array of nanoparticles (NPs) used in agriculture, ZnO stands out for its ability to alleviate salt stress across plant species. It has been employed in numerous studies where it positively alleviated salt stress, including *Oryza sativa* L (Singh et al., 2022), *Brassica napus* L (El-Badri et al., 2021), *Triticum aestivum* L (Adil et al., 2022), *Lycopersicon esculentum* L (Faizan et al., 2021a), *Hordeum vulgare* L (Ali et al., 2022). and *Vicia faba* L (Mogazy et al., 2022). Zinc (Zn) is an essential mineral with numerous roles in plant growth and development (Burman et al., 2013; Farooq et al., 2021; Nasrallah et al., 2022). It is involved in the maintenance of membrane structure, chlorophyll biosynthesis, cell division, and the activity of enzymes, including RNA and DNA polymerase (Davarpanah et al., 2016; Abbasifar et al., 2020; Vaghar et al., 2020).

Based on the literature, the uptake and transport of NPs can be influenced by particle size, concentration, exposure time, surface charge, and plant species. Different application modes, such as foliar application, nano priming, and soil application, can also influence the NPs uptake and transport within the plant system (Dilnawaz et al., 2023). For example, in the foliar application, the NPs enters the plant system through stomata, cuticle penetration, hydathodes, and leaf wounds (Singh et al., 2018). In the case of soil application, the NPs enter through the symplastic pathway, apoplastic pathway, lenticel, and injuries (Singh et al., 2018). In contrast, in nano-priming, the NPs can enter the seeds through a class of ubiquitous membrane proteins known as aquaporins, which are involved in transporting water and other solutes (Wohlmuth et al., 2022). Thus, selecting the appropriate application method for NPs in plants is critical for achieving optimal results.

*Phaseolus vulgaris* L., commonly known as bush beans, is a widely cultivated legume valued for its high nutritional content. However, salt stress significantly hampers its growth and productivity, given its high sensitivity to salinity (Garcia et al., 2019). Despite advancements in NPs mediated stress alleviation, the potential of ZnO NPs to mitigate salt stress in *P. vulgaris* remains largely unexplored. Moreover, previous studies on ZnO NPs in *P. vulgaris* primarily focus on enhancing plant growth and productivity, leaving a notable gap in understanding their role in salt stress mitigation. Therefore, this study aims to assess the salt stress mitigating potential of ZnO NPs in *P. vulgaris* through three distinct modes of application: foliar application, nano-priming, and soil application. Specifically, the objectives include evaluating the effect of ZnO NPs on the physiological and biochemical attributes of *P. vulgaris* under salt stress conditions and identifying the optimal concentration of ZnO NPs application across these three modes to enhance plant resilience.

## 2 Materials and methods

### 2.1 Plant growth conditions and experimental setup

Common bush bean (*Phaseolus vulgaris* L.; BLANCHE, yellow 68204) seeds were collected from MORAVOSEED CZ, Czech Republic. Seeds were sterilized using a 1% (*v/v*) solution of commercial bleach-Savo (NaClO) for 10 minutes and subsequently washed thoroughly with distilled water several times (Bharati et al., 2023a). Afterward, the seeds were placed on a paper towel to dry until they reached their natural moisture level.

The sterilized seeds were potted in plastic pots (11×11×23 cm) filled with substrate containing a light peat mixture (KLASMANN TS2, Czech Republic). For the first 48 hours, they were kept in the dark to promote germination. Afterward, the growth chamber was set with a controlled environment with 285 µmolm^-2^s^-1^ (PPFD), a photoperiod of 16 light/8 dark hours, and a temperature of 25°C during light and 22°C during the dark phase with a relative humidity of 65-70%. Plants were left to grow until the first true leaves were grown. It took around 10 days to attain a full-grown true leaf. The experimental setup comprised 14 treatments to evaluate the effects of ZnO nanoparticles and salt stress on plant growth. The treatments included a control group, a salt stress group (200 mM NaCl), and combinations of salt stress with ZnO nanoparticle applications via priming (P), foliar spray (F), and soil application (S) at four different concentrations (25 mg/L, 50 mg/L, 100 mg/L, and 200 mg/L). ZnO nanoparticle solutions (Sigma-Aldrich, <50 nm) were prepared using double-distilled water and dissolving using a BANDALIN SONOPLUS sonicator.

Salt stress was induced by adding 15 ml of a 200 mM NaCl solution to the soil in each pot. The optimal salt concentration was determined from preliminary germination tests (**Supplementary Table S1**). ZnO nanoparticle treatments were administered in the above-mentioned three modes. For foliar spray, 15 ml of ZnO nanoparticle solution was sprayed onto each salt-stressed plant at the specified concentration. For soil application, 15 ml of ZnO nanoparticle solution at the specified concentration was added to the soil of each salt-stressed plant. For seed priming, sterilized seeds were primed with ZnO nanoparticle solutions at the specified concentrations for six hours with occasional shaking (Ul Haq et al., 2023). The primed seeds were then rinsed with distilled water, dried to their natural moisture level, planted, and grown to the true leaf stage before being subjected to salt stress.

ZnO nanoparticle and salt solutions were applied together every alternative day for two weeks, consisting of seven applications. All treatments were replicated five times and arranged in a completely randomized design. Water levels in the pots were monitored throughout the experiment using double distilled water. However, irrigation was avoided on treatment days. No additional nutrients were provided to the plant during the experiment.

### 2.2 Nanoparticle characterization

X-ray diffraction (XRD) patterns were produced for the nano powder by high-resolution transmission electron microscopy (HRTEM) using the FEI Talos F200X microscope operating at 200 kV. A microscopic copper grid covered by a holey carbon film was used as specimen support for TEM investigations.

### 2.3 Plant growth analysis

Morphological growth parameters such as fresh weight, dry weight, plant height, root length, and leaf thickness of randomly selected plants were measured to assess the effect of salinity stress and nanoparticle applications.

### 2.4 Pigments contents

To calculate total chlorophyll and carotenoids, leaf cuts (using cork borer) of randomly selected young leaves were macerated in 1 mL N, N-Dimethylmethanamide for six hours in the dark. After that, the solutions were kept on the horizontal shaker for 30 minutes, and then the solution was run on a spectrophotometer (Evolution 201, Thermo Scientific) at 663 nm, 646 nm, and 480 nm for chlorophyll a, b, and carotenoids. Total chlorophyll content was measured by adding chlorophyll a and b. MultispeQ was used to measure relative chlorophyll content (SPAD values).

### 2.5 Leaf relative water content

The leaf relative water content (LRWC) was measured by leaf cuts of fresh leaves of beans plants; firstly, the fresh weight (FW) of leaves was measured using an electrical weighing balance, then leaves were kept in distilled water for at least four hours then turgid weight (TW) was recorded. After that, leaves were placed in a drying oven at 60°C, and the oven dry weight (DW) was determined, then LWRC was calculated using the below-mentioned formula (Mullan and Pietragalla, 2012).


$$\text{RWC}(\%)=\frac{\text{FW}-\text{DW}}{\text{TW}-\text{DW}}\times 100$$

### 2.6 Photosynthetic parameters

To assess fluorescence-based photosynthetic parameters, electrochromic shifts, and photosynthetic assessment were done using a MultispeQ V 2.0 device, which is linked with the PhotosynQ platform (http://www.photosynq.org). Parameters like linear electron flow (LEF), Proton conductivity of the thylakoid membrane (gH^+^), non-photochemical excitation quenching (ϕNPQ), non-regulatory energy dissipation(ϕNO), non-photochemical quenching (qN), photochemical quenching (qP), steady- state proton flux (vH^+^), and QA redox state (q_L_), PSI active centers and PSI over reduced centers were observed (Bharati et al., 2023b).

### 2.7 Proline content

The proline content (µg/g fresh weight of tissue) was measured using the (Bates et al., 1973) protocol. Leaf tissue (0.5g) was homogenized with glacial acetic acid in mortar and pestle and then filtered through filter paper. Then, the filtrate was mixed with an equal volume of ninhydrin and placed in a water bath at 90°C for 30 minutes. After that, the samples were allowed to cool down completely to arrest the reaction. Then, toluene was added to each tube and placed on a shaker for 30 minutes. Blank was used to set the spectrophotometer; absorbance was taken at 520 nm.

### 2.8 Malondialdehyde content

The total malondialdehyde (MDA) content was quantified using the thiobarbituric acid (TBA) assay with slight modifications to the published protocol (Du and Bramlage, 1992). Briefly, leaf samples were homogenized in liquid nitrogen, extracted, and filtered through filter paper. 700 µL of ethanolic extract was mixed with 700 µL of 0.6% TBA and 10% trichloroacetic acid (TCA). The mixture was then heated in a water bath at 95°C for 25 minutes, cooled to room temperature, and centrifuged at 11,000 g for one minute in a Hermle Z216 MK centrifuge. The absorbance of the supernatant was measured at 600, 532, and 440 nm against blank.

### 2.9 Total phenolic content

To quantify the total phenolic content (TPC), an aliquot of ethanolic extract was mixed with a 10-fold diluted Folin-Ciocalteau reagent and 7% sodium carbonate to prepare a reaction mixture. The volume was adjusted to 2.5 mL using ultrapure water (Singleton and Rossi, 1965), allowed to react for 90 min, then the absorbance of the samples was measured at 765 nm against the blank using a UV/VIS spectrophotometer (Evolution 201, Thermo Scientific). The TPC was calculated as gallic acid equivalents (mg/g FW) used for the calibration curve.

### 2.10 Hydrogen peroxide assay

For the determination of H_2_O_2_ in plant tissues, an adapted method (Junglee et al., 2014) was used. Leaf samples (100 mg) were homogenized with 1 mL of solution containing 0.25 mL (0.1% w/v) trichloroacetic acid (TCA), 0.5 mL potassium iodide (KI) (1 M) and 0.25 mL (10 mM) potassium phosphate buffer (pH 7) at 4°C. The homogenate was centrifuged at 12,000 g for 15 min at 4°C. One tube with H_2_O instead of KI is used for the tissue coloration background for blanks. 200µL of supernatant from each tube was taken and placed at room temperature for 20 minutes of incubation. Samples and blanks (without plant extract) were prepared in triplicates. The absorbance was measured at 350 nm.

### 2.11 Total protein content

Leaf tissue (100 mg) was grounded with liquid nitrogen and extracted with 1 mL of phosphate buffer (50 mM; pH 7). The homogenate was centrifuged at 10,000 rpm, 4°C for 20 minutes. The protein concentration was determined using the Bradford method (Bradford, 1976), which used a spectrophotometer and bovine serum albumin as a standard for the construction of the calibration curve. The absorption of samples was measured at 595 nm, and the extraction buffer was used as blank.

### 2.12 Antioxidant enzyme assay

For the determination of enzyme activity, phosphate extract was prepared. Catalase (CAT) activity was adapted according to (Lück, 1965) protocol. The supernatant was mixed with H_2_O_2_ and 50 mM phosphate buffer (pH 7.0), and a change of absorbance was measured at 240 nm. 1 unit of CAT activity was determined as the decrease 0.01 A_240_ unit min^-1^. A previously described method was used for the estimation of guaiacol peroxidase activity (POD) (MAEHLY and CHANCE, 1954). Briefly, 50 mM of phosphate buffer (pH 7.0) was mixed with guaiacol and H_2_O_2,_ and this mixture was added to the protein extract. The absorbance was immediately measured at 420 nm. 1 unit of POD activity was determined as the 0.01 A420 unit min-1 increase. Superoxide dismutase (SOD) activity was measured according to adapted (Giannopolitis and Ries, 1977) method. The reaction solution consisted of H_2_O, 50 mM phosphate buffer (pH 7.8), L-methionine, Triton-X, NBT, and protein extract or extraction buffer as blank. The reaction was started by adding riboflavin, and the samples were kept under light for 15 minutes. Incubation was stopped by putting the samples in the dark, and absorbance was measured at 560 nm. 1 unit of SOD activity was considered as the ability to inhibit NBT photo-reduction for 50%.

### 2.13 Quantification of Na, Zn, and other elements

Approximately 600mg of dried samples were weighed and placed in a quartz vessel, and 4 mL of HNO_3_ (Analpure^®^, Analytika, Czech Republic) and 2.0 mL of H_2_O_2_ (Rotipuran^®^, Carl Roth, Germany) were added. The prepared samples were then digested in a closed vessel microwave system for 20 mins at 180°C. The digested solutions were transferred to 50 mL polypropylene tubes and filled with Milli-Q water (≥ 18.2 MΩ cm^-1^; MilliQ system, Millipore, SAS, France) up to a final volume of 45 mL. Then, the elemental concentration of some macro and micro elements was analyzed by an inductively coupled plasma mass spectrometry (ICP-MS; Agilent 7700×, Agilent Technologies Inc., USA). A certified reference material, namely peach leaves, was included for quality assurance (SRM-1547, NIST). Three biological and three technical replicates were taken for each genotype.

### 2.14 Statistical analysis

The statistical analysis of all procured data was done by one-way ANOVA (analysis of variance), using Tukey’s honest significant difference *post hoc* comparison test. The significant difference was used at a 0.05% level of significance. Principal component analysis was done using Graph Pad Prism version 10.

## 3 Results

### 3.1 Characterization of zinc oxide nanoparticles

**Figure 1:**
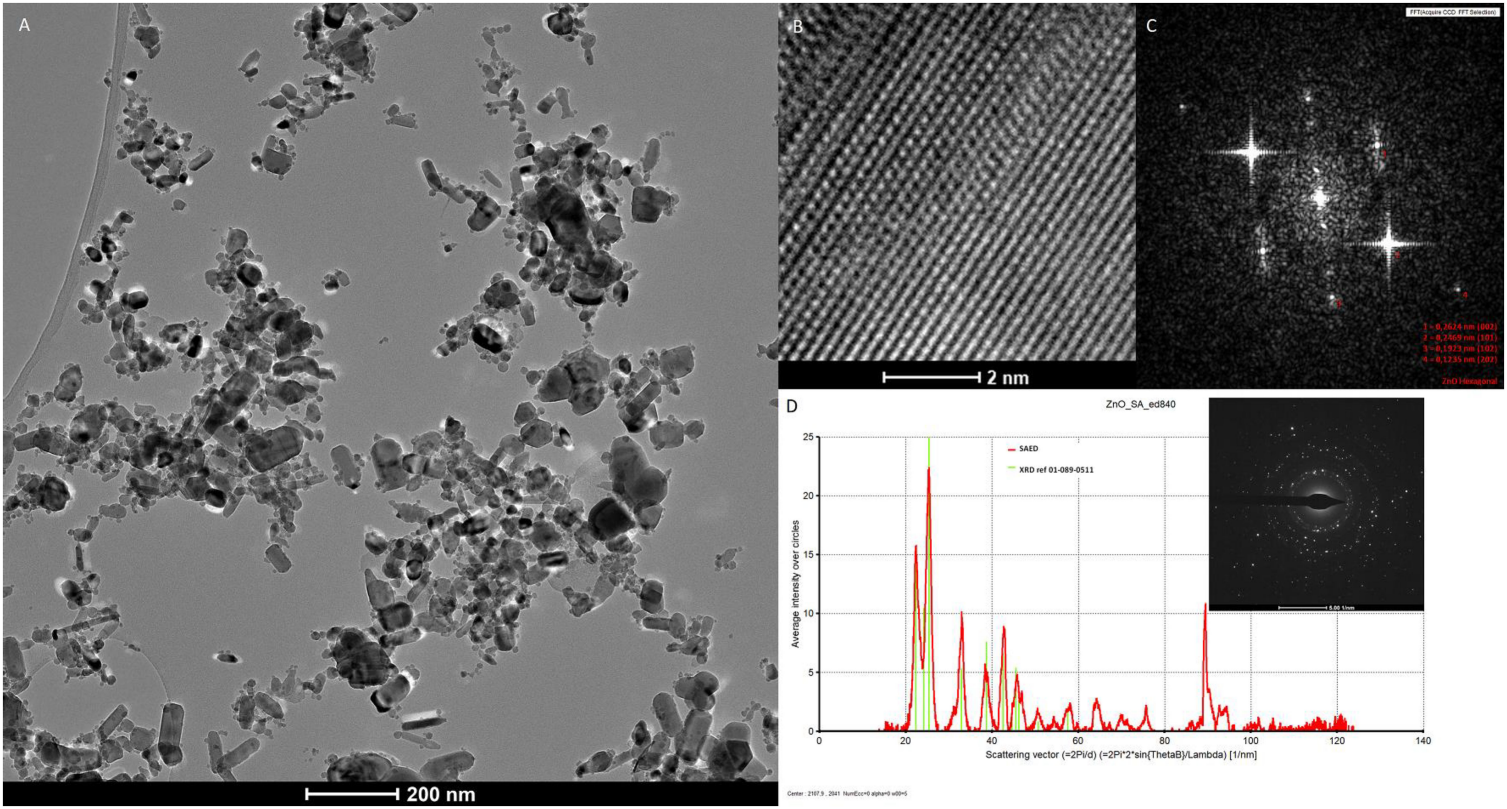
**(A)** TEM micrograph of ZnO nanoparticles shows broad particle size distribution; **(B)** High-resolution transmission electron micrograph (HRTEM) of hexagonal structure of ZnO; **(C)** FFT of HRTEM micrograph, most intensive reflections can be indexed to the (002), (101), (102) and (202) planes of ZnO hexagonal phase; **(D)** Selected area electron diffraction (SAED) pattern processed using process. The diffraction program and SAED data are in very good agreement with XRD reference data of cubic of ZnO (01-089-0511).

Transmission electron microscopy (TEM), X-ray diffraction (XRD), and selected area electron diffraction pattern (SAED) were used to determine the form and real size of the ZnO NPs. XRD planes corresponding to the (002), (101), (102), and (202) plane indicated typical Zinc Oxide crystals. The hexagonal rod-shaped ZnO has an average length of 87.52 nm and a width of 39.76 nm (**Figure 1**).

### 3.2 Morphological parameters

**Figure 2:**
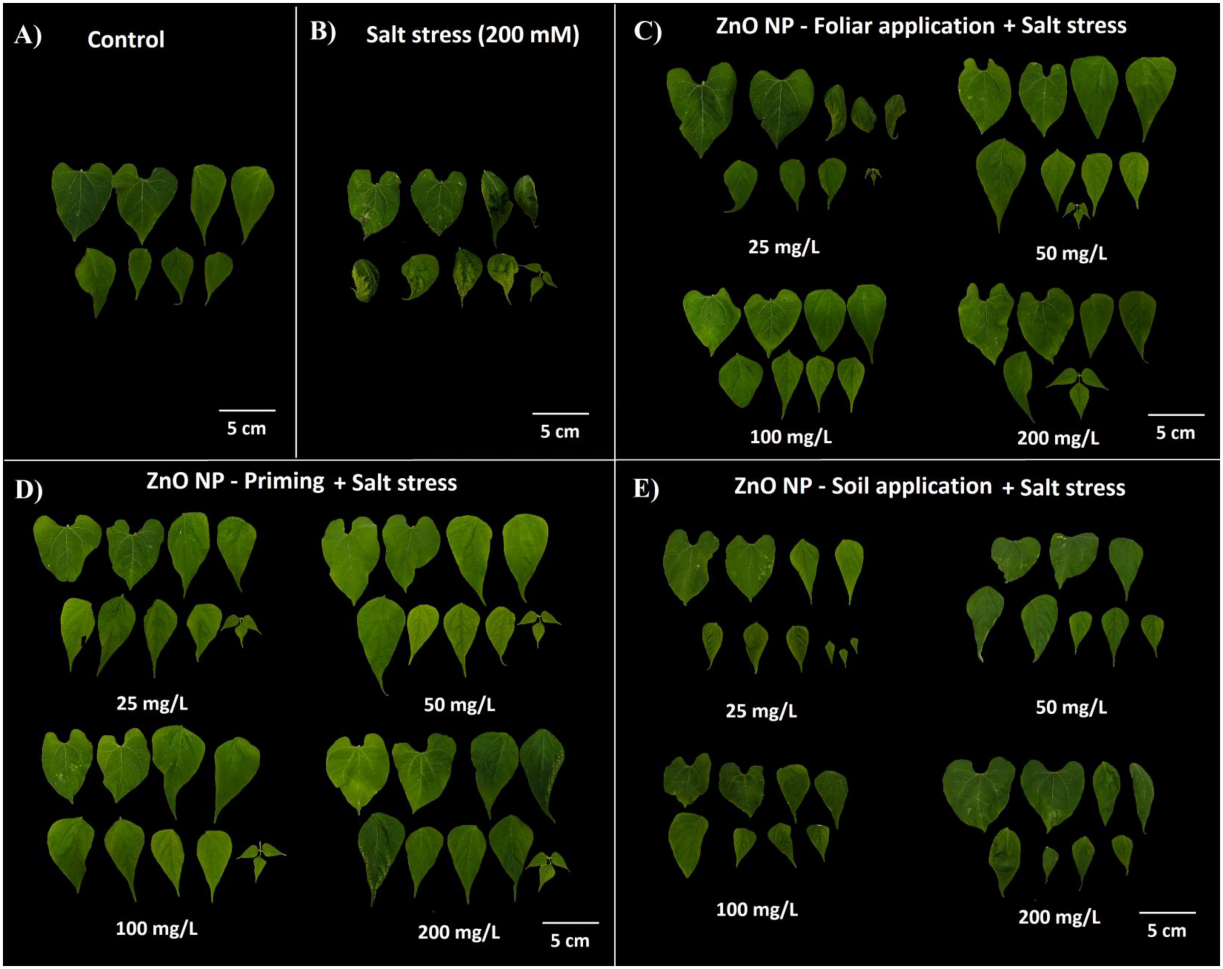
Leaf morphology of plants under different treatments; **(A)** control; **(B)** salt treatment; **(C)** foliar application of ZnO NPs with salt stress; **(D)** ZnO NPs priming with salt stress; **(E)** soil application of ZnO NPs with salt stress.

The impact of salinity stress on bean plants was investigated, revealing a notable reduction in fresh weight by approximately 24% compared to control plants (**Table 1**). However, applying ZnO NPs led to a significant improvement in fresh weight, increasing it by 39-83% in foliar application, 60-68% in nano-priming, and 31-55% in soil application compared to salt-stressed plants. Similarly, dry weight analysis further underscored the beneficial effect of NPs, demonstrating a significant increase of 30-52% in foliar application, 52-92% increase in nano-priming, and 52-56% increase in soil application treatment compared to salt-treated plants. The salt stress caused a 27.13% reduction in dry weight compared to the control plants. Salt stress also significantly affected relative water content among leaves and was reduced by 27% compared to the control plants. All modes of ZnO NPs application effectively augmented the relative water content in leaves. Specifically, foliar application elevated it by 50-78%, nano-priming by 40-75%, and soil application by 27-41%. Regarding plant height, salt-treated plants experienced a notable decrease in height by 33% compared to the control plants. However, nano-priming exerted a substantial influence, enhancing plant height by 59-92%. In contrast, other application modes, such as foliar application and soil treatment, exhibited no significant changes compared to salt-stressed plants. Conversely, salt stress did not significantly affect root length and was comparable with the control plants. However, moderate levels of ZnO NPs through foliar application and nano-priming significantly augmented the root growth compared to control plants. Interestingly, salt-treated plants demonstrated a significant augmentation, exhibiting a 2.4-fold increase in leaf thickness compared to control plants. Similarly, plants subjected to NP treatment through soil application also displayed a substantial increase, ranging between a 2 to 2.3-fold change in leaf thickness relative to the control group. However, other treatments, including foliar application and priming, yielded leaf thickness values comparable to control plants (**Table 1**; **Figure 2**).

**Table 1:** Effect of different concentrations and modes of ZnO nanoparticle application on plant morpho-physiological parameters.

Treatments	Fresh weight (mg)	Dry weight (mg)	Relative water content (%)	Plant height (cm)	Root length (cm)	Leaf thickness (mm)
**Control**	32.8 ± 2.51cde	4.57 ± 0.25bc	70.47 ± 9.6bcd	70.2 ± 12.57bcd	30.35 ± 4.84ef	0.26 ± 0.1fg
**Salt**	24.9 ± 4.6f	3.33 ± 0.12d	51.4 ± 5.93e	47.35 ± 11.4e	26.8 ± 5.98f	0.63 ± 0.22a
**25 mg/L F**	38.73 ± 1.43abcd	4.63 ± 0.15bc	83.13 ± 10.16abc	56.25 ± 18.58de	32.32 ± 3.23cde	0.41 ± 0.18cdefg
**50 mg/L F**	45.63 ± 3.58a	5.07 ± 0.61ab	91.25 ± 1.21a	62.45 ± 17.73bcde	35.65 ± 3.08bcd	0.44 ± 0.19abcdef
**100mg/L F**	34.83 ± 1.1bcd	5.03 ± 0.21ab	77.33 ± 6.26abc	61.65 ± 19.85cde	38.48 ± 2.82ab	0.37 ± 0.19defg
**200 mg/L F**	34.5 ± 2.12bcd	4.33 ± 0.15bc	76.92 ± 3.95abc	55.95 ± 13.61de	31.38 ± 2.23cdef	0.36 ± 0.12efg
**25 mg/L P**	39.87 ± 5.47abcd	5.07 ± 0.68ab	90.09 ± 2.83ab	90.97 ± 13.43a	42.57 ± 2.36a	0.22 ± 0.07g
**50 mg/L P**	41.6 ± 1.08ab	5.17 ± 1.07ab	78 ± 9.7abc	81.39 ± 8.95ab	36.22 ± 1.54bc	0.38 ± 0.12defg
**100 mg/L P**	41.73 ± 3.22ab	5.4 ± 0.95ab	78.47 ± 12.18abc	78.48 ± 10.54abc	32.96 ± 1.27cde	0.43 ± 0.11bcdefg
**200 mg/L P**	40.8 ± 1.21abc	6.4 ± 0.2a	72.15 ± 1.56abcd	75.09 ± 10.56abcd	33.3 ± 2.12cde	0.51 ± 0.17abcde
**25 mg/L S**	38.8 ± 1.31abcd	5.13 ± 0.31ab	65.17 ± 2.16cd	57.15 ± 13.62de	30.6 ± 5.48def	0.53 ± 0.1abcde
**50 mg/L S**	34.9 ± 2.85bcd	5.2 ± 0.4ab	68 ± 5.29cd	61.05 ± 15.68cde	30.46 ± 4.28ef	0.56 ± 0.06abcd
**100 mg/L S**	34.43 ± 1.95bcd	5.2 ± 0.53ab	72.53 ± 6.06abc	58.3 ± 8.38de	29.24 ± 4.38ef	0.58 ± 0.1abc
**200 mg/L S**	32.73 ± 4.01de	5.07 ± 0.85ab	69.26 ± 13.85cd	61.38 ± 7.26cde	28.84 ± 3.12ef	0.61 ± 0.13ab

Values represent mean ± SD, and different letters show significant differences among treatments at 0.05% of significance.

### 3.3 Pigment content

Relative chlorophyll content was assessed to elucidate the effects of salt stress. A significant decrease of 20% was observed in salt-treated plants’ SPAD values compared to control plants (**Table 2**). Conversely, the application of NPs yielded no significant differences relative to both stressed and control plants. Similarly, salt stress resulted in a substantial reduction of 37% in total chlorophyll content compared to control plants, with no discernible differences observed in total chlorophyll content following NPs application relative to salt-stressed plants, except for low to moderate levels of NPs applied via foliar and soil application. Specifically, foliar application of NPs led to an increase in chlorophyll content ranging from 27% to double that of salt-stressed plants. Nano-priming exhibited no significant change relative to salt-stressed plants, while soil application markedly enhanced chlorophyll content by 47-57%. Similarly, carotenoid content was also afflicted by salinity stress and displayed a decline of 43.6% compared to control plants. However, applying NPs exhibited a promising trend in mitigating this decline. Specifically, foliar application of moderate NPs (50 and 100 mg/L) significantly enhanced carotenoid content by 66% to 90%. Moreover, at a higher concentration of nano priming (200 mg/L), carotenoid levels showed a notable increase of 47%. Conversely, soil application of NPs yielded no significant difference compared to salt-stressed plants (**Table 2**).

**Table 2:** Effect of different concentrations and modes of ZnO nanoparticle application on pigments.

Treatments	Chl a (µ mol.m^-2^)	Chl b (µ mol.m^-2^)	Total chlorophyll (µ mol.m^-2^)	Carotenoids (µ mol.m^-2^)	SPAD Value
**Control**	4626.27 ± 567.96b	1530.57 ± 186.53abcd	6156.84 ± 754.05b	959.74 ± 147.78ab	42.13 ± 6.63a
**Salt**	2924.86 ± 99.87c	963.74 ± 34.7e	3888.6 ± 133.78c	540.37 ± 16.72e	33.67 ± 4.97b
**25 mg/L F**	3740.67 ± 227.15bc	1197.92 ± 77.19cde	4938.59 ± 303.68bc	708.15 ± 25.41cde	36.75 ± 5.11ab
**50 mg/L F**	4778.88 ± 799.39b	1715.7 ± 334.52ab	6494.58 ± 1132.38ab	898.75 ± 128.56abc	37.19 ± 2.95ab
**100mg/L F**	6054.25 ± 2039.21a	1928.87 ± 664.44a	7983.12 ± 2703.49a	1027.9 ± 339.15a	37.11 ± 2.73ab
**200 mg/L F**	3839.72 ± 362.54bc	1326.77 ± 126.35bcde	5166.49 ± 487.34bc	618.35 ± 54.82de	36.9 ± 3.29ab
**25 mg/L P**	2957.38 ± 336.52c	947.26 ± 104.9e	3904.65 ± 434.05c	554.07 ± 55.49e	33.85 ± 3.66b
**50 mg/L P**	3974.68 ± 179.28bc	1278.39 ± 73.09cde	5253.08 ± 249.94bc	653.03 ± 37.89de	36.02 ± 6.85ab
**100 mg/L P**	4021.8 ± 209.96bc	1327.54 ± 80.68bcde	5349.34 ± 290.25bc	712.65 ± 40.99cde	35.81 ± 3.87ab
**200 mg/L P**	3865.27 ± 358.54bc	1218.81 ± 131.04cde	5084.08 ± 488.39bc	793.11 ± 63.81bcd	33.95 ± 4.14b
**25 mg/L S**	4504.21 ± 216.3b	1613.41 ± 218.07abc	6117.06 ± 337.21b	760.81 ± 81.65bcde	37.4 ± 2.83ab
**50 mg/L S**	4414.59 ± 558.53b	1319.25 ± 115.66bcde	5733.79 ± 986.2b	730.18 ± 40.53cde	38.98 ± 2.77ab
**100 mg/L S**	4109.72 ± 127.44bc	1189.18 ± 59.17de	5298.61 ± 834.19bc	684.14 ± 83.9cde	36.16 ± 4.07ab
**200 mg/L S**	3843.22 ± 212.29bc	1249.06 ± 49.71cde	5092.21 ± 446.86bc	666.49 ± 132.98de	36.63 ± 3.68ab

Values represent mean ± SD, and different letters show significant differences among treatments at 0.05% of significance.

### 3.4 Photosynthetic parameters

**Figure 3:**
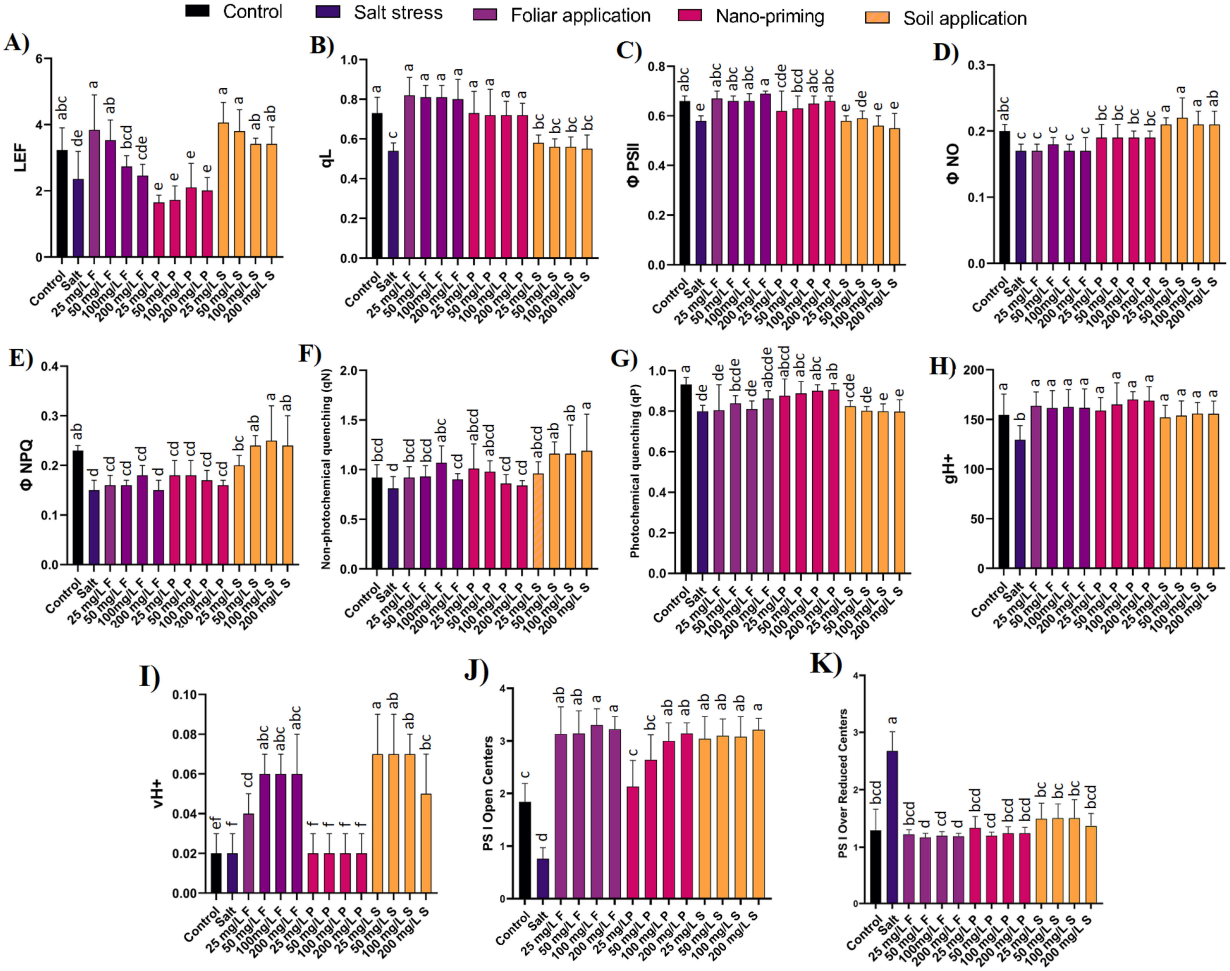
Effect of different concentration and modes of ZnO NPs application on fluorescence-based photosynthetic parameters **(A)** LEF, **(B)** qL, **(C)** ϕ PSII, **(D)** ϕNO, **(E)** ϕNPQ, **(F)** qN, **(G)** qP **(H)** gH^+^, **(I)** vH^+^, **(J)** PS I open centres, and **(K)** PS I over reduced centres. Bar values show mean value and error bars show standard deviation. Different letters on the bar show statistical significance at level 0.05%.

Linear electron flow (LEF) is a process that occurs during the light-dependent reactions of photosynthesis. It involves the flow of electrons through a series of protein complexes and molecules in the thylakoid membrane of chloroplasts. The primary purpose of LEF is to generate ATP and NADPH, which are used in the Calvin cycle to produce carbohydrates. Linear electron flow also showed a significant decline of 27% in the presence of salt compared to control plants. Lower concentrations of foliar application of ZnO NPs (25, 50 mg/L) significantly increased electron flow by 63% and 49%, respectively, compared to salt-stressed plants. Nano priming did not show any significant difference, while the soil application of ZnO NPs increased electron flow by 44% to 72% compared to salt-stressed plants (**Figure 3A**).

The qL parameter quantifies the extent of state transitions occurring within the photosynthetic apparatus, providing valuable insights into the dynamic regulation of photosynthetic light harvesting and photoprotection mechanisms. qL parameter significantly decreased by 26% in comparison to control plants. The foliar application of ZnO NPs significantly increased qL by 48% to 51% compared to salt-stressed plants. Nano priming also elevated qL by 33% to 35% compared to salt-stressed plants. However, soil application of ZnO NPs did not significantly differ in qL values compared to salt-treated plants (**Figure 3B**).

Φ PSII, the quantum efficiency of PSII decreased by 12% in salt-stressed plants compared to control plants. In ZnO NPs treatment through the foliar application, all the tested concentrations significantly increased by 16% to 18%; in nano priming, an increase of 9% to 14% was observed, while all concentrations of ZnO NPs through soil treatment and lower concentrations of nano-priming (25 mg/L) does not show any significant difference when compared with salt-stressed plants (**Figure 3C**).

Φ NO was not found to be statistically significant between the control and salt-stressed plants. Similarly, all the modes of application of ZnO NPs did not exhibit any significant difference, except for soil treatment of NPs, where it showed an increase of 29% compared to salt-stressed plants (**Figure 3D**). Φ NPQ showed a significant 35% drop in salt-stressed plants compared to control plants, and the drop was consistent among all the concentrations of the foliar and nano-priming mode of application of NPs, where the values remained statistically insignificant compared to salt-stressed plants. However, ZnO NP application through soil treatment displayed an increase in Φ NPQ values by 33-67% compared to salt-stressed plants (**Figure 3E**). However, the non-photochemical quenching (qN) that protects plants from damage caused by excess light energy did not exhibit any difference in the salt-stressed plants compared to control plants. In contrast, photochemical quenching (qP) was found to decrease significantly in salt-stressed plants compared to control plants. Only priming with zinc oxide was found to be effective in maintaining photochemical quenching at levels comparable to the control (**Figures 3F, G**).

The proton conductivity (gH^+^) in plants is primarily required for the function of the proton pump complexes in the thylakoid membranes of chloroplasts during photosynthesis. gH^+^ decreased by 16% in salt-treated plants compared to control plants. NPs application significantly improved this parameter. In foliar application, an increase of 24% to 26% was observed; in nano priming, an increase of 22% to 31% was observed; and in soil applications, an increase of 17% to 20% was observed (**Figure 3H**).

The vH^+^ parameter in photosynQ provides valuable insights into the dynamic regulation of steady sate proton flux employed by photosynthetic organisms to optimize photosynthetic efficiency. vH^+^ has not shown any significant difference between control plants and salt-treated plants. However, foliar and soil application of ZnO NPs significantly increased this parameter by 100% to 200% and 150% to 250%, respectively, compared to salt-treated and control plants. Nano priming of ZnO NPs showed no significant difference between salt-treated and control plants (**Figure 3I**).

The impact of salt stress on the Photosystem I (PSI) light-harvesting capacity was substantial. Under salt stress conditions, the open PSI center showed a significant reduction of 58.6% compared to control plants, indicating a marked decrease in the plant’s ability to harvest light efficiently. In line with this, the PSI-reduced active center exhibited an increase of 51.9% compared to control plants, further establishing the adverse effects of salinity on PSI functionality. The application of ZnO NPs demonstrated a promising mitigation strategy against the detrimental effects of salt stress on PSI activity. Both foliar and soil applications of ZnO NPs were effective in managing the PSI active center. Specifically, these treatments alleviated the stress-induced reductions in PSI functionality and contributed to maintaining higher levels of PSI activity under salt stress conditions (**Figures 3J, K**).

### 3.5 Biochemical analysis

**Figure 4:**
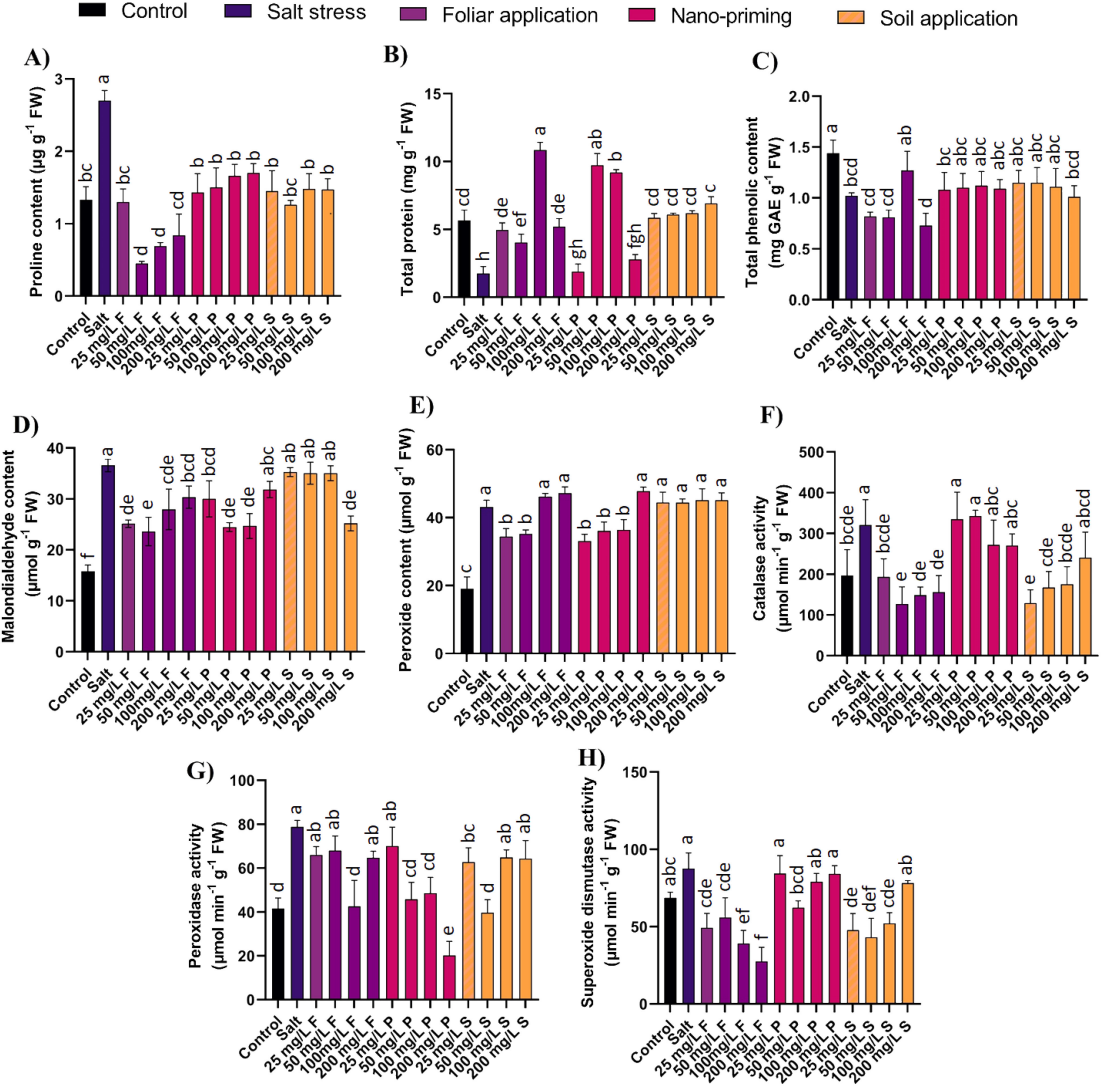
Effect of different concentrations and modes of ZnO NPs application on biochemical parameters **(A)** Proline, **(B)** Total protein content, **(C)** TPC, **(D)** MDA, **(E)** H_2_O_2_, **(F)** CAT, **(G)** POD, **(H)** SOD. Bar values show mean value and error bars show standard deviation. Different letters on the bar show statistical significance at level 0.05%.

Proline, an evident mark of osmotic stress, accumulated more than 100% in salt-stressed plants compared to the control plants. All the modes of ZnO NPs application were effective in reducing proline accumulation. Foliar application of ZnO NPs was found effective in decreasing the cellular accumulation of proline by 52% to 83%; ZnO nano priming and soil application were also potent in reducing the proline accumulations by 37%-47% and 45-53%, respectively (**Figure 4A**).

Proteins are very important elements of cells as structural and functional parts with a wide spectrum of activities in the form of various enzymes. Salinity stress reduces protein formation in plants; results show a decline of 69% in salt-stressed plants compared to control plants. ZnO NPs application significantly supported protein production; foliar application increased their amount by 3 folds to 6 folds, and nano priming increased protein production ranging from 5 folds to 6 folds at moderate concentrations (50, 100 mg/L). The level of proteins was comparable with that of the control after NPs application (**Figure 4B**).

Phenolic compounds are a crucial component in maintaining plant growth and managing stresses by plants. Salinity reduced the total phenolic content by 29% in the salt-stressed plants compared to the control plants. ZnO NP application in all the application modes did not exhibit any significant difference in total phenolic content compared to salt-stressed plants (**Figure 4C**).

MDA accumulation nearly doubled in salt-stressed plants, marking the salinity stress and cell membrane damage. ZnO NPs application was found to be effective in decreasing the MDA accumulation. Specifically, foliar applications decreased MDA accumulation by 17% to 35%, and nano priming at lower to moderate concentrations (25-100 mg/L) reduced it by 17%-33%. Soil application effectively decreased MDA content (by 31%) only at higher concentrations (200 mg/L) (**Figure 4D**).

In plants under stress conditions, production of hydrogen peroxide is often elevated (e.g., through detoxification of superoxide), and its role as one of the signal molecules can lapse, and it participates in the creation of other reactive oxygen species (ROS) (Smirnoff and Arnaud, 2019). In the presence of salt stress, the peroxide content in plants increased by more than two-fold compared to control plants. However, when foliar applications of ZnO NPs were administered at lower concentrations (25 and 50 mg/L), there was a reduction in peroxide production by 18% to 20%. Similarly, nano-priming with ZnO NPs at lower to moderate concentrations (25-100 mg/L) decreased peroxide content by 16% to 20%. Interestingly, higher concentrations of foliar application (100 and 200 mg/L) and nano-priming (200 mg/L) showed no significant difference compared to control plants. Additionally, all concentrations of ZnO NPs applied through soil treatment did not significantly reduce peroxide content and remained comparable to levels observed in salt-stressed plants (**Figure 4E**).

CAT is an antioxidant enzyme that combats surplus H_2_O_2_ to reduce ROS toxicity; it was highly active in salt-stressed plants and increased by 63% compared to control plants. ZnO NPs foliar application reduced the salinity stress severity indicated by the reduced CAT production by 39% to 60%. Similarly, soil applications of ZnO NPs significantly decreased CAT production ranging from 45% to 60%. However, the ZnO nano priming did not display any significant difference compared to salt-stressed plants (**Figure 4F**).

POD is another enzyme that acts against oxidative stress. POD concentration was found to have increased significantly by 90% in salt-stressed plants compared to control plants. The amount of POD was decreased under foliar application of ZnO NPs, especially at a specific concentration of 100 mg/L, where the concentration was reduced by 46%. Nano priming also affected this enzyme (except at 25 mg/L) by decreasing POD by at least 38% and up to 74%. Soil application of ZnO NPs, significantly manifested at lower concentrations (25, 50 mg/L) and reduced it by 20% to 50% (**Figure 4G**).

SOD is a very effective enzyme as it converts superoxide into oxygen and hydrogen peroxide. In response to salt stress, the SOD content shot up by 27% in salt-stressed plants compared to control plants. However, the foliar application of ZnO NPs reduced the SOD presence significantly by 36% to 68%, and lower to moderate concentrations (25-100 mg/L) of ZnO NPs in soil application decreased SOD production by 40% to 51%. Nano-priming of ZnO was effective at a specific concentration of 50 mg/L (**Figure 4H**).

### 3.6 ICP-MS

Salinity stress is known to influence plants’ nutrient uptake significantly. This study examined the nutrient profiles of both macro and micronutrients in plant shoot and root samples.

#### 3.6.1 Nutrient homeostasis in shoot and root

**Figure 5:**
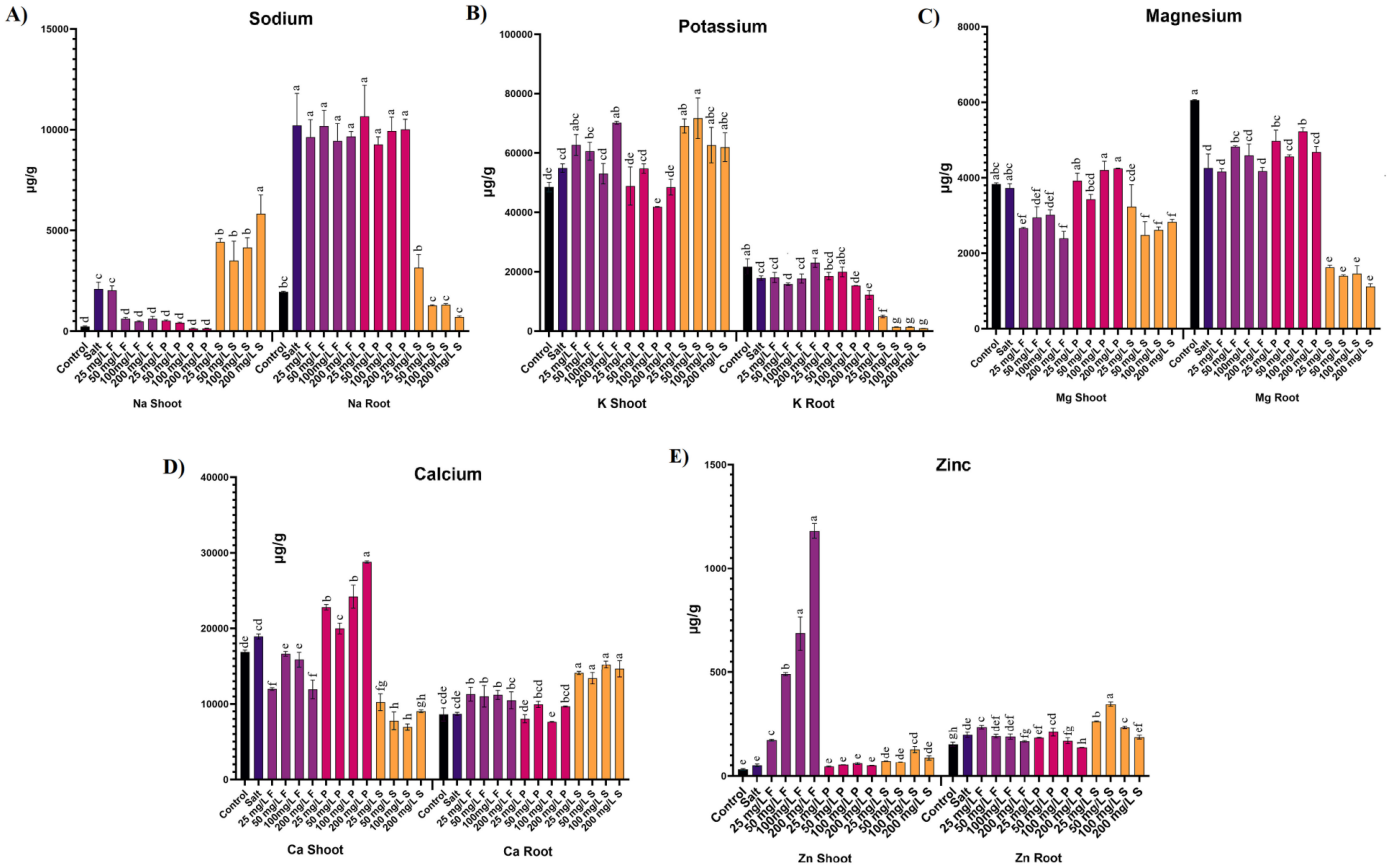
Effect of different concentrations and modes of ZnO NPs application on ion uptake **(A)** Sodium, **(B)** Potassium, **(C)** Magnesium, **(D)** Calcium, **(E)** Zinc. Bar values show mean value and error bars show standard deviation. Different letters on the bar show statistical significance at level 0.05%.

Salt stress induced a remarkable accumulation of sodium (Na) in the aerial parts of plants, reaching nearly 9-fold higher levels compared to control plants. However, applying ZnO NPs significantly reduced Na accumulation in aerial parts. Specifically, all concentrations of ZnO NPs via foliar application and nano-priming led to reductions in Na accumulation by 70-77% and 75-94%, respectively. Conversely, soil application of ZnO NPs failed to decrease Na concentration; instead, it increased significantly by 1.6 to 2.7-fold (**Figure 5D**). Similarly, Salt stress caused a significant increase in the Na accumulation by more than 5 folds in roots. Foliar applications and nano-priming of ZnO NPs, both modes of application at all the tested concentrations, were ineffective in reducing the higher Na concentration in the roots, and the values were comparable to the salt-stressed plants. However, the soil application of ZnO NPs effectively reduced the Na accumulation in the root by 69-93% across the tested concentrations (**Figure 5D**).

Potassium (K) levels showed insignificant differences between the aerial parts of control and salt-stressed plants. Notably, a specific concentration of 100 mg/L of ZnO NPs through nano-priming led to a 24% reduction in K concentration. Conversely, lower concentrations (25, 50 mg/L) of ZnO NPs via soil treatment and a higher concentration (200 mg/L) of foliar application significantly increased K accumulation by 26%, 30%, and 28%, respectively (**Figure 5A**). K was also reduced in the salt-treated plant roots by 18%, although a higher concentration of 200 mg/L of Foliar increased the potassium concentration by 29%. On the contrary, the higher concentration (200 mg/L) of nano-priming significantly reduced the K concentration by 32%. Following a similar trend, all the tested concentrations of ZnO NPs through soil application reduced the K concentrations by 72-95% (**Figure 5A**).

Magnesium (Mg) concentration did not significantly differ between control and salt-stressed plants. Similarly, nano-primed plants exhibited comparable Mg accumulation to control and salt-stressed plants. However, foliar application and soil treatment of ZnO NPs showed a significant decline in Mg accumulation, except for the lower concentration (25 mg/L) of soil application, which did not differ from salt-stressed plants. Specifically, foliar application reduced Mg accumulation by 19-36%, while soil application reduced it by 24-33% (**Figure 5C**). Likewise, Salt stress induced a significant reduction of 30% in Mg levels in roots. Nevertheless, specific concentrations of foliar application (50 mg/L) and nano-priming (50,100 mg/L) exhibited augmented Mg levels in roots. In contrast, all tested concentrations of ZnO NPs through soil application reduced Mg concentration by 62 to 74% (**Figure 5C**).

Calcium (Ca) concentration between control and salt-stressed plants did not differ significantly. However, applying ZnO NPs through foliar application and soil treatment led to a significant decline in Ca concentration by 12 to 37% and 46 to 63%, respectively. Interestingly, ZnO NPs nano-priming at all concentrations increased Ca accumulation by 20-52%, except for the 50 mg/L concentration treatment, which did not differ from salt-stressed plants (**Figure 5B**). While Ca remained unaffected by salt stress in roots, its levels increased significantly in treatments where ZnO NPs were applied through foliar and soil treatment.

Zinc (Zn) levels showed no significant differences between control and salt-stressed plants. However, foliar application of ZnO NPs resulted in a substantially higher accumulation of Zn in aerial parts, increasing by 3 to nearly 24-fold compared to salt-stressed plants. Additionally, a specific concentration of 100 mg/L of ZnO NPs treatment via soil application increased Zn accumulation by 2.5-fold compared to salt-stressed plants. Nano-priming with ZnO NPs and other concentrations of soil application did not significantly differ from control and salt-stressed plants (**Figure 5E**). Conversely, Zn experienced a significant increase of 29% under salt stress in roots, further escalating by 20% at lower concentrations (25 mg/L) of ZnO NP foliar application compared to salt-treated plants. Notably, higher concentrations of ZnO NPs through foliar application (200 mg/L) and nano-priming (100, 200 mg/L) yielded lower accumulations of Zn in roots compared to salt-stressed plants. However, soil application of ZnO NPs at lower to mid concentrations demonstrated higher accumulations of Zn in the roots of treated plants (**Figure 5E**). A detailed table is available in the **Supplementary Material** (**Supplementary Table S2**).

### 3.7 Evaluation of correlation between measured parameters using PCA analysis

PC score and loading plot using 14 different treatments and 38 measured parameters were prepared. The eigenvalues (**Supplementary Table S3**) of the correlation matrix indicated that there were 13 principal components (PC), which showed a 100% correlation of data. PC 1 and PC 2 exhibited 31.68% and 29.18% variability within data, respectively. Therefore, they explained a total variance of 60.86%. According to the PCA scores, the ZnO NP mode of applications was distinctly clustered, with priming closely clustered with the control, whereas foliar and soil application formed entirely different clusters.

**Figure 6:**
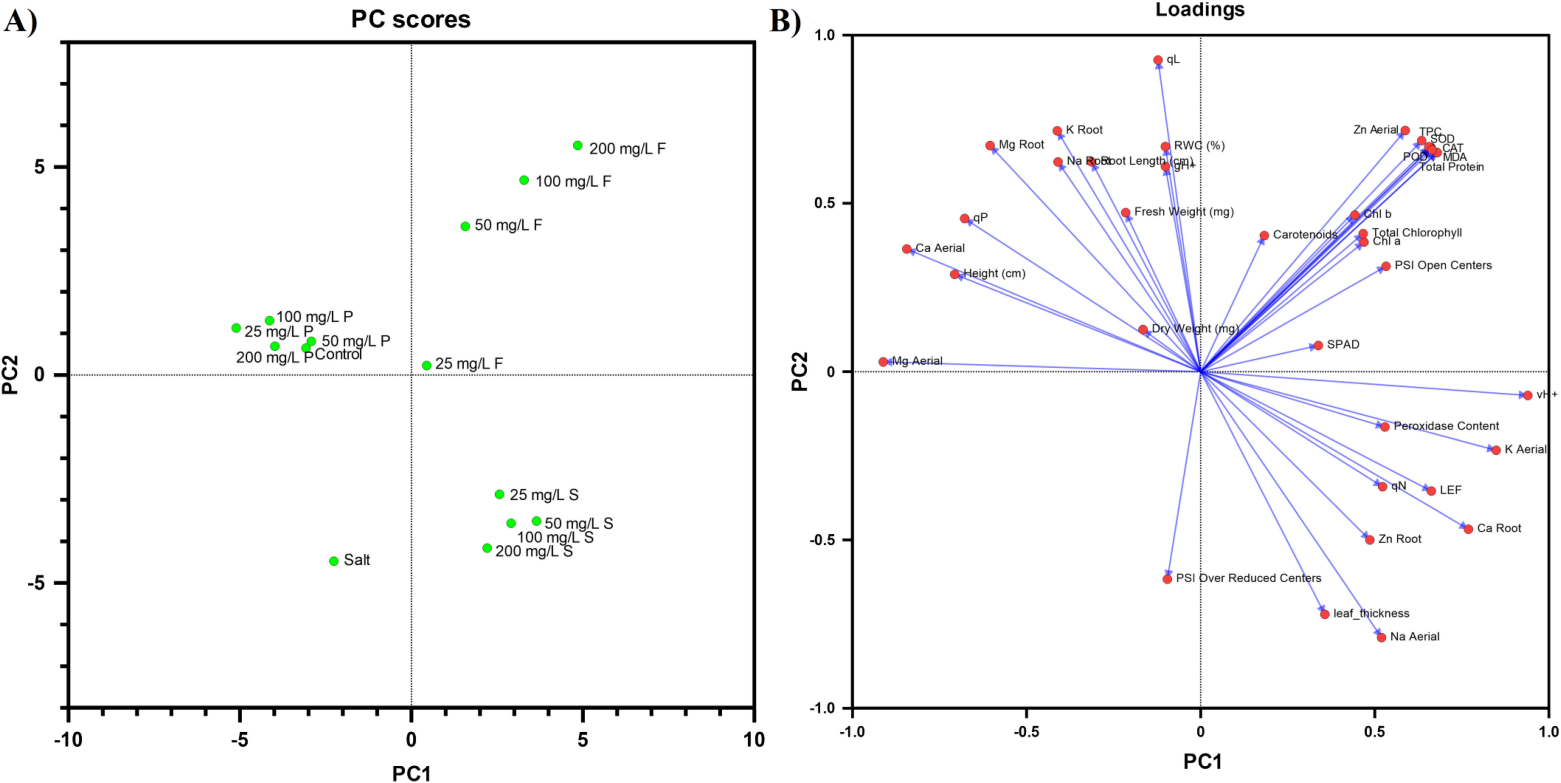
The principal component of the analysis of measured parameters is represented by **(A)** treatments and **(B)** variables. PC 1 and PC 2 exhibited 31.68% and 29.18% variability respectively, a total variance of 60.86%.

According to PCA loadings, the parameters including vH+, POD, peroxidase content (H_2_O_2_), K shoot, LEF, qN, Ca root, Zn root, leaf thickness, are Na shoot are positively correlated to PC1 while negatively correlated to PC2. Additionally, parameters such as Mg shoot, Mg root, Ca shoot, height, qP, K root, Na root, fresh weight, dry weight, root length, qL, gH+, and RWC are positively correlated to PC2 while negatively correlated to PC1. Some parameters such as Zn shoot, total protein, SOD, POD, CAT, MDA, Chl a, Chl b, total chlorophyll, carotenoids, SPAD, and PSI open centers were found to be positively correlated to both PC1 and PC2. The parameter PS I over reduced centers was found to be negatively correlated to both PC 1 and PC 2 (**Figure 6**).

## 4 Discussion

The detrimental effects of salinity stress on plant growth and productivity are well-documented, posing significant challenges to agricultural sustainability and food security. Salt hampers water uptake and osmotic potential, leading to cell turgidity loss. Salinity also hampers pigments in plants, further disrupting photosynthetic efficiency and leading to loss of plant growth (Jajoo, 2013; Wu et al., 2014). Furthermore, salinity produces reactive oxygen species due to the impairment of different physicochemical reactions, which disrupts cell integrity and function (Munns and Tester, 2008). Sodium accumulation also leads to disturbance in plant ion homeostasis, which adversely affects many channel proteins and leads to an imbalance in ion uptake, causing yield loss (Jajoo, 2013). To mitigate salt stress, among many measures, ZnO NP application is found effective in many plants (Mahdieh et al., 2018; El-Badri et al., 2022; Mogazy et al., 2022). The nanoparticle introduction method is also playing a significant role in its effectiveness. In this study, foliar application, priming, and soil application of ZnO NPs were tested to observe the mitigation of salinity stress in bean plants (Salehi et al., 2023).

Bean plants were severely affected when subjected to salinity stress. Many physiological changes were prominent in salt-stressed plants, such as reduced fresh weight, dry weight, and relative water content, indicating water imbalance in plants. Results from (Zia-ur-Rehman et al., 2023) show a similar pattern of osmotic imbalance, loss in fresh weight, and RWC in wheat plants. In the current study, ZnO NP application through all application modes effectively mitigated the water imbalance in salt-stressed plants. This suggests that ZnO NPs may enhance the ability of bean plants to maintain water balance and mitigate the negative impacts of salt stress on growth. Exogenous application of ZnO NPs is known for improving osmotic imbalance in plants by promoting the synthesis of antioxidants and enzymatic activity and regulating carbohydrate metabolism when plants are exposed to saline stress (Sun et al., 2021). Consequently, this contributes to enhancements in biochemical and physiological responses, such as maintaining water equilibrium, increasing the accumulation of compatible solutes, and safeguarding cells against reactive oxygen species (Li et al., 2021).

The salt-stressed plants displayed shunted growth with reduced shoot and root length. The studied morphological parameters were improved upon ZnO NPs application, particularly nano-priming and foliar application. Previous studies employing ZnO NPs through foliar application and nano-priming effectively increased salt tolerance in tomato and *Zea mays*, respectively (Faizan et al., 2021b; Mohammadi et al., 2023). The current results also align with previous studies on green peas and moringa (Guha et al., 2018). These effects can be attributed to the imperative role of Zn in protein synthesis, chlorophyll synthesis, and protection of biological membrane integrity by reducing excess Na^+^ and Cl^-^ ions (Faizan et al., 2021b).

The observed reduction in chlorophyll and carotenoid content in response to salinity stress highlights the detrimental impact of this abiotic stressor on the photosynthetic pigment composition of plants. Chlorophyll and carotenoids play crucial roles in photosynthesis and photoprotection, and their decline under salt stress conditions can severely impair plant growth and productivity (Zahra et al., 2022). The current study’s findings align with previous studies demonstrating the susceptibility of chlorophyll and carotenoid pigments to disruption under saline conditions (Taïbi et al., 2016), resulting in diminished photosynthetic efficiency and compromised plant performance (Qados, 2010). However, the application of NPs presented a promising avenue for mitigating the adverse effects of salinity stress on chlorophyll and carotenoid levels. Despite falling short of control values, NP treatment led to significantly higher pigment concentrations than salt-stressed plants alone. The foliar application was found most effective in maintaining chlorophyll and carotenoid content, which is supported by (Mahawar et al., 2024), who found that ZnO foliar application maintains pigments in salinity stress conditions.

Salinity stress significantly adversely affected key fluorescence-based photosynthetic parameters, disrupting crucial processes such as electron transport, energy storage, and photoprotection mechanisms (Wu et al., 2014; Faizan et al., 2021b). Linear electron flow (LEF), a crucial process in photosynthesis for ATP and NADPH generation, was significantly impaired by salinity stress in this study. The foliar application and soil treatment of ZnO NPs showed promising results in increasing LEF. Similar observations were previously made where authors reported that the ZnO NPs improve water splitting and improve electron flow (Rai-Kalal and Jajoo, 2021). Similarly, foliar application and priming were able to maintain the quantum yield (ϕ PSII) in salt-stressed plants approximately to the levels of control plants (Mahawar et al., 2024). According to Elshoky et al. (2021), this could be attributed to the ability of ZnO NPs to enhance stomatal activity in plants, which might improve the quantum yield of photosystem II. Likewise, foliar application and nano priming observed increased proton conductivity (gH^+^) and steady-state proton flux (vH^+^). Proton conductivity and steady-state proton flux are crucial parameters that maintain the proton gradient in the thylakoid membrane and produce energy currencies continuously to maintain different physiological processes in plants (Guo et al., 2019; Khatri and Rathore, 2019). According to (Rai-Kalal and Jajoo, 2021), ZnO NP priming influences the increase in the reaction center in leaves, which leads to an increase in electron transport and proton conductivity in wheat. PSI activity was also significantly influenced by the treatment of ZnO NPs; it protected the reaction center by over-reducing in salt stress (Elshoky et al., 2021).

Results of the current study provide valuable insights into the biochemical responses of bean plants to salinity stress and the potential mitigating effects of ZnO nanoparticle treatments on various stress indicators and antioxidant systems. Total protein content, crucial for plant building blocks and defense mechanisms, declined in salt-treated plants, indicating disruptions in protein synthesis and function under salinity stress. NPs application, primarily through foliar application and nano priming, significantly increased protein content, suggesting a restoration of protein synthesis and potential enhancement of defense mechanisms against stress. A similar increase in the protein content in the presence of ZnO NP application was reported by (Tavallali et al., 2021), indicating their potential to enhance protein levels or mitigate protein degradation under salinity stress conditions. Similarly, proline is an amino acid that acts as an osmoregulator in plant cells and manages osmotic fluctuation (Hayat et al., 2012). The salt stress induced a significantly higher proline accumulation in the current study, indicating an increased osmotic imbalance. However, ZnO NPs application displayed a significant decrease in proline accumulation irrespective of mode of application, highlighting the potential of ZnO NPs in mitigating the osmotic stress caused by salinity. Many results support our result, where ZnO NPs are effective in reducing proline accumulation by decreasing stress levels (Alabdallah and Alzahrani, 2020; Singh et al., 2022).

ROS production directly participates in the overproduction of MDA, which causes oxidative stress by impairing the cell membrane integrity of the bean plants under salt stress (Rizwan et al., 2019; Fatemi et al., 2021). In our study, we found MDA showing a downward trend in the presence of ZnO NPs in all three treatments. In contrast, some concentrations of foliar application and nano priming effectively reduced peroxide content compared to salt-stressed plants. A previous study by (Foroutan et al., 2019) reported similar results, where ZnO NPs treatment effectively protected cell membrane integrity by reducing the MDA contents in *Moringa peregrina* (Forssk.) Fiori. These results demonstrate ZnO NPs application supports ROS scavenging activity and oxidative stress mitigating potential in bean plants.

Oxidative stress in plants causes increased production of antioxidant enzymes such as SOD, POD, and CAT (Sharma and Dubey, 2005; Smirnoff and Arnaud, 2019). Many studies have shown that NPs application enhances antioxidants to manage salinity stress (Schützendübel and Polle, 2002). Antioxidant enzyme assays revealed complex responses of catalase (CAT), peroxidase (POD), and superoxide dismutase (SOD) to salinity stress and NPs treatments. While salt stress-induced increases in CAT and POD content, NPs treatments led to varied responses in enzyme activity levels. Foliar application and soil treatment of ZnO effectively restored CAT and POD content to control levels, while POD remained unaffected. However, higher concentrations of nano-priming and lower concentrations of soil application resulted in the reduction of SOD accumulation. Previously, nano-priming in wheat yielded a similar role of ZnO NPs in reducing enzymatic activities, including SOD, POD, and CAT. Studies suggest that the decreased antioxidant enzymatic activities might be caused by the reduction of ROS formation (Mahawar et al., 2024).

The role could also play non-enzymatic antioxidants here, like carotenoids or various phenolic compounds that participate in defense reactions. As was described in a study focused on canola, the application of zinc NPs improved the content of these metabolites under salt stress (Farouk and Al-Amri, 2019).

Salinity stress causes significant elemental imbalance, leading to ion toxicity in plants (Singh et al., 2018). It was evident from the ICP-MS results that Na^+^ accumulation in the salt-stressed plants was significantly higher than in control plants. The higher accumulation of Na^+^ is known to impede the uptake of other crucial nutrients, adversely affecting plant growth (Assaha et al., 2017). Na^+^ accumulation in roots was unaffected by foliar and priming treatment and remained comparable to salt-stressed plants. However, it reduced significantly in the aerial part of the plants in both foliar and priming applications. Plants often regulate other nutrients like K^+^ or Ca^2+^ to decrease the accumulation of Na^+^ (Bacha et al., 2015). Correspondingly, the plants treated with ZnO NPs through foliar application displayed significant Ca^2+^ accumulation in roots, whereas nano-priming promoted higher accumulation of Ca^2+^ in the aerial parts. Plants are well known to exhibit a rapid increase in calcium concentrations under salt stress (Manishankar et al., 2018). This suggests that the ZnO NPs promoted the accumulation of higher calcium concentrations in plants to combat the excessive Na^+^.

Similarly, soil application of ZnO NPs also displayed a significant decrease in Na^+^ accumulation with a sharp increase of Ca^2+^ in the roots. Interestingly, Na^+^ was significantly higher in the plants’ areal part than in all the other treatments. This could be attributed to the increased permeability of cell membranes or altered ion transport processes facilitated by NPs (Schwab et al., 2016).

In summary, our results highlight the substantial potential of ZnO nanoparticles (NPs) as a sustainable approach to alleviate salinity stress and boost the resilience of *P. vulgaris* under high salinity conditions. Our comprehensive PCA analysis indicates that nano-priming with ZnO NPs yields a response similar to that of control plants for the parameters assessed, positioning it as a superior method for mitigating salt stress in bean plants compared to the other mode of application used. Nano-priming offers additional advantages for large-scale agriculture due to its cost-effectiveness and efficiency, making it an attractive option for broader applications. In contrast, while foliar application and soil treatment are practical, they are better suited for smaller farms due to their labor-intensive nature and sensitivity to environmental conditions such as heavy rain and high temperatures. Furthermore, these methods may leave residual nanoparticles, which could have potential long-term effects (Tourinho et al., 2012). While the beneficial effects of nanoparticles on plant growth are well-documented, it is crucial to consider possible negative impacts. Previous research has highlighted concerns about nanoparticles potentially decreasing chlorophyll synthesis, impairing photosynthetic performance, and affecting plant antioxidant activities.

Moreover, concerns exist regarding their impact on soil microbiota and potential risks to human health (Rasheed et al., 2022). Considering this, future research should focus on field trials to evaluate these application methods in real-world agricultural settings, which will help confirm and expand upon the findings of this study. These insights provide a foundation for future investigations and practical applications in agricultural sustainability.

## 5 Conclusion

In conclusion, this study elucidates for the first time the potential of Zinc Oxide (ZnO) NPs in alleviating salinity stress in common beans. Through a comprehensive analysis encompassing physiological, biochemical, and nutrient parameters, we demonstrated ZnO NPs’ efficacy in mitigating salinity stress’s detrimental effects on bean plants. Specifically, ZnO NPs have been observed to diminish oxidative stress by reducing the accumulation of proline, malondialdehyde (MDA), and hydrogen peroxide while affecting the activities of pivotal antioxidant enzymes such as catalase (CAT), peroxidase (POD), and superoxide dismutase (SOD). These findings underscore the capacity of ZnO NPs to modulate antioxidant defense mechanisms, thereby attenuating oxidative damage and improving plant growth under salinity stress conditions. Furthermore, ZnO NPs exhibit promise in regulating ion uptake and accumulation, thereby influencing nutrient homeostasis. Notably, ZnO NPs have effectively reduced sodium (Na) accumulation in aerial parts while facilitating the uptake of calcium (Ca), consequently contributing to enhanced nutrient balance and overall plant growth. Based on the PCA analysis, nano-priming with ZnO NPs was the most effective among the tested application methods. In summary, our findings underscore the significant potential of ZnO NPs as a sustainable solution for mitigating salinity stress and enhancing the resilience of *P. vulgaris* L. to elevated saline conditions. Future research should focus on elucidating the underlying mechanisms governing ZnO nanoparticle-mediated stress tolerance and exploring their broader applicability in diverse crop species to address the prevailing challenges in global agriculture.

## Data Availability

The original contributions presented in the study are included in the article/**Supplementary Material**. Further inquiries can be directed to the corresponding author.
