# Supplementary material for: Zinc oxide nanoparticles application alleviates salinity stress by modulating plant growth, biochemical attributes and nutrient homeostasis in Phaseolus vulgaris L
Source: Front Plant Sci. 2024 Sep 4;15:1432258. doi: 10.3389/fpls.2024.1432258 (PMC11408239; doi:10.3389/fpls.2024.1432258)
Supplement: Supplementary file 1 [file Table1.docx]

**Table S1.** Rate of seed germination in different salt concentrations

| Control (%) | 25mM Salt (%) | 50 mM Salt (%) | 100 mM Salt  (%) | 200 mM salt  (%) |
| --- | --- | --- | --- | --- |
| 86.7±10.3a | 78.3±11.7a | 75.2±10.5a | 71.7±15.7ab | 65.2±12.5b |

Values represent mean ±SD, and different letters show significant differences among treatments at 0.05% of significance.

| **Treatments** | **K Shoot** | **Ca Shoot** | **Mg Shoot** | **Na Shoot** | **Zn Shoot** |
| --- | --- | --- | --- | --- | --- |
| **Control** | 48538.47±1565.53de | 16852.57±263.81de | 3832.43±34.28abc | 233.83±30.55d | 31.22±3.91e |
| **Salt** | 54913.9±1448.81cd | 18925.92±336.18cd | 3723.57±119.86abc | 2101.27±337.80c | 49.96±6.14e |
| **25 mg/L F** | 62714.76±3511.34abc | 11973.1±175.58f | 2662.79±24.36ef | 2032.77±221.97c | 171.26±3.4c |
| **50 mg/L F** | 60610.28±3001.88bc | 16633.47±309.54e | 2941.13±295.71def | 623.06±70.93d | 490.19±7.66b |
| **100 mg/L F** | 53002.86±3446.99cd | 15853.1±975.47e | 3014.84±139.87def | 483.39±35.66d | 685.72±79.79a |
| **200 mg/L F** | 70203.9±493.23ab | 11934.68±1233.85f | 2388.23±190.82f | 619.05±118.24d | 1179.43±35.36a |
| **25 mg/L P** | 48861.93±6419.54de | 22798.04±357.19b | 3922.29±198.7ab | 516.85±41.18d | 44.81±2.24e |
| **50 mg/L P** | 54744.93±1591.7cd | 19985.51±713.37c | 3431.79±131.67bcd | 416.9±34.53d | 52.5±0.33e |
| **100mg/L P** | 41870.8±142.89e | 24201.95±1514.69b | 4207.18±232.07a | 127.96±36.11d | 59.18±4.82e |
| **200mg/L P** | 48484.94±2640.67de | 28796.82±143.66a | 4244.67±10.5a | 134.49±15.97d | 50.08±0.83e |
| **25 mg/L S** | 69091.69±2376.1ab | 10245.18±1111.64fg | 3236.85±579.29cde | 4425±167.58b | 71.09±0.86de |
| **50 mg/L S** | 71715.17±6847.69a | 7778.16±1171.53h | 2479.51±357.59f | 3504.14±966.01b | 65.98±0.82de |
| **100 mg/L S** | 62647.91±5976.12abc | 6941.56±410.66h | 2613.63±81.85ef | 4145.59±490.29b | 127.34±14.48cd |
| **200 mg/L S** | 61956.45±4849.54abc | 9049.08±175.12gh | 2822.8±75.83def | 5823.75±945.02a | 86.94±10.97de |
| **Treatments** | **K Root** | **Ca Root** | **Mg Root** | **Na Root** | **Zn Root** |
| **Control** | 21699.10±2643.38ab | 8616.26±868.66cde | 6056.14±11.57a | 1950.76±33.59bc | 151.92±10.56gh |
| **Salt** | 17888.33±744.45cd | 8673.45±216.76cde | 4260.54±369.90d | 10218.60±1578.42a | 196.73±12.28de |
| **25 mg/L F** | 18094.93±1709.79cd | 11304.73±904.17b | 4159.75±80.97d | 9631.91±869.97a | 236.16±8.36c |
| **50 mg/L F** | 15815.14±403.23d | 11022.03±1419.76b | 4825.84±22.17bc | 10181.22±780.32a | 190.64±8.7def |
| **100 mg/L F** | 17718.75±1536.77cd | 11202.01±597.37b | 4594.70±301.57cd | 9446.57±858.33a | 188.04±12.19def |
| **200 mg/L F** | 23066.15±1574.16a | 10491.52±1113.00bc | 4173.73±102.58d | 9661.77±252.82a | 166.26±3.26fg |
| **25 mg/L P** | 18544.58±1239.16bcd | 8044.00±544.22de | 4975.13±288.99bc | 10655.26±1549.53a | 183.39±2.24ef |
| **50 mg/L P** | 19966.23±1625.69abc | 9944.41±410.06bcd | 4564.71±45.66cd | 9268.19±380.23a | 211.25±20.06cd |
| **100mg/L P** | 15353.37±37.82de | 7635.04±52.04e | 5232.05±96.69b | 9943.89±675.26a | 168.85±13.49fg |
| **200mg/L P** | 12214.76±1429.93e | 9683.74±70.68bcd | 4682.20±147.53cd | 10013.09±502.48a | 136.83±0.55h |
| **25 mg/L S** | 4960.03±434.31f | 14097.90±211.22a | 1623.34±57.68e | 3154.98±650.84b | 263.49±1.79b |
| **50 mg/L S** | 1402.09±52.79g | 13403.58±755.19a | 1402.27±24.06e | 1279.32±32.58bc | 345.48±10.88a |
| **100 mg/L S** | 1410.76±72.08g | 15199.05±437.05a | 1461.81±208.90e | 1315.48±57.95bc | 235.05±5.44c |
| **200 mg/L S** | 901.10±9.27g | 14651.18±1077.33a | 1116.05±75.52e | 708.83±48.76c | 185.52±9.26def |

**Table S2.** Effect of different modes of ZnO nanoparticle application on ion uptake behavior in root and shoot of bean plants. Measuring units was µg/g.

Values represent mean ±SD, and different letters show significant differences among treatments at 0.05% of significance.

**Table S3.** Principal component analysis (PCA) of studied variables along with their eigenvectors

| Variables | PC1 Eigenvectors | PC 2 Eigenvectors | PC 3 Eigenvectors |
| --- | --- | --- | --- |
| leaf_thickness | 0.104174917403407 | -0.219420045976341 | 0.0518212630962262 |
| LEF | 0.193499618090533 | -0.107997405070467 | -0.135460168592208 |
| qN | 0.152592193723332 | -0.104064471301308 | -0.188316581923411 |
| qP | -0.197974449821735 | 0.138435207078419 | -0.0752245403685176 |
| qL | -0.035770397163931 | 0.281776234236184 | -0.0324334691156412 |
| vH+ | 0.274451189119026 | -0.0215455949375256 | -0.0836098329452851 |
| gH+ | -0.0295994678024915 | 0.185509516971864 | -0.250600443033839 |
| PSI Open Centers | 0.15552035370999 | 0.0952412735833761 | -0.248159276858248 |
| PSI Over Reduced Centers | -0.0279294329139967 | -0.187673542146607 | 0.284781431043564 |
| SPAD | 0.0987282798900536 | 0.023597064559692 | -0.173946494960133 |
| Fresh Weight (mg) | -0.0628922342864324 | 0.143719013340949 | -0.254489440008738 |
| Dry Weight (mg) | -0.0483650380739703 | 0.0380532617829306 | -0.332869429045799 |
| RWC (%) | -0.0296024961749987 | 0.203469373635831 | -0.163179114836605 |
| Height (cm) | -0.206367219286236 | 0.0879481586513183 | -0.173939479962323 |
| Root Length (cm) | -0.0914235974011911 | 0.189707646930894 | -0.113392714029634 |
| Chl a | 0.137010179688788 | 0.117201917902686 | -0.22403328838252 |
| Chl b | 0.129348728105523 | 0.141562470938859 | -0.190545014900622 |
| Total Chlorophyll | 0.136319683633986 | 0.124754507830181 | -0.217419054738584 |
| Carotenoids | 0.0534090887266102 | 0.123042625693073 | -0.235605590713197 |
| Na Root | -0.119642954894573 | 0.189635254589266 | 0.173363758642856 |
| Na Aerial | 0.151741191886946 | -0.240457943580634 | -0.0367763319841503 |
| Mg Root | -0.176622047920392 | 0.204425823578082 | 0.0786341127514359 |
| Mg Aerial | -0.266263712226624 | 0.00879447500763522 | -0.0116185011737592 |
| K Root | -0.120259631849614 | 0.217679671200991 | 0.175333767876125 |
| K Aerial | 0.247880606818022 | -0.0710826513573027 | 0.0381538978857209 |
| Ca Root | 0.224631641295705 | -0.142440055849727 | -0.138902396488615 |
| Ca Aerial | -0.24652449092216 | 0.110879450132446 | 0.0276654515345461 |
| Zn Root | 0.141942657756803 | -0.152171864567094 | -0.0827711665620522 |
| Zn Aerial | 0.171583227187477 | 0.217983913813246 | 0.145470293082824 |
| MDA | 0.19833084742359 | 0.198110463272073 | 0.135251546257316 |
| Proline | 0.19833084742359 | 0.198110463272073 | 0.135251546257316 |
| TPC | 0.185397225802148 | 0.208814274433893 | 0.140858013918113 |
| Total Protein | 0.19328317949278 | 0.202569694518429 | 0.137609608425331 |
| Peroxidase Content | 0.154538065352058 | -0.0500567673028314 | 0.0887256768746196 |
| CAT | 0.193005014826911 | 0.202121363543766 | 0.138826024586997 |
| POD | 0.191347607134566 | 0.203262068220139 | 0.139855369955408 |
| SOD | 0.194089059010188 | 0.200539862588012 | 0.13931805309972 |
